# Efficient Polar Coded Selective Decode-and-Forward with Cooperative Decision Threshold in Cooperative Multi-Relay Transmissions

**DOI:** 10.3390/s23010165

**Published:** 2022-12-24

**Authors:** Bin Jiang, Yue Tang, Jianrong Bao, Chao Liu, Yanhai Shang

**Affiliations:** 1 School of Communication Engineering, Hangzhou Dianzi University, Hangzhou 310018, China; 2 School of Electronics and Information, Hangzhou Dianzi University, Hangzhou 310018, China; 3 Sichuan Provincial Energy Investment Group Co., Ltd., Chengdu 610041, China; 4 National Mobile Communications Research Laboratory, Southeast University, Nanjing 210096, China

**Keywords:** cooperative communications, polar coding, selective decode-and-forward, cooperative decision threshold, channel state information

## Abstract

In some satellite Internet of Things (IoT) devices with terrain shielding, the qualities of the direct source-destination (S-D) channel are poor, requiring cooperative communications with multi-relays to be employed. In order to solve error propagation of current decode-and-forward (DF) on such occasions, an efficient polar coded selective decode-and-forward (SDF) cooperation method is proposed with a new decision threshold derived from channel state information (CSI). First, the proposed threshold is derived from the CSI by exploiting the channel gain ratio of optimal relay-destination link (R-D) with source-relay (S-R) link. The above R-D link possesses good channel quality among all links in the system. Second, when the channel gain ratio of certain relay links is larger than the aforementioned decision threshold, the source and all these relays cooperatively send messages together to the destination to accomplish perfect SDF transmission. Otherwise, all relays are frozen and the messages are directly transmitted through the S-D link. If it fails anyway, a retransmission is subsequently tried in the next transmission cycle. In addition, a polar code for fading channels is designed and adaptively adjusted to a proper code rate according to channel quality to attain good bit error rate (BER) performance. Simulation results show that the proposed scheme achieves about 0.9 and 0.5 dB gain at BER of $10^{-4}$, respectively, in multi-relay cooperative communications with multi-path fading channels compared with those of non-cooperation and existing polar coded cooperation channels. Therefore, the proposed polar coded SDF (PCSDF) scheme can improve both the BER and the outage probability (OP) performance in multi-relay cooperative systems, making it quite suitable for heterogeneous network applications in cooperative satellite IoT systems involving sixth-generation (6G) communications.

## 1. Introduction

Multiple-input multiple-output (MIMO) represents a milestone in wireless cooperative communications, bringing efficient transmission rates and reliability. Today, virtual MIMO uses relays as virtual antennas [1], and has been widely used and rapidly developed. In cooperative communications, a typical system mainly includes three components, e.g., a source, relays, and a destination. Diversity gain is obtained when the relay nodes forward messages and the destination node combines all received signals from both the source and relay nodes. Recently, there have been several cooperative schemes proposed, such as amplify-and-forward (AF) [1], decode-and-forward (DF) [2], coded cooperation (CC) [3], and more. Traditionally, cooperative transmission has brought about several benefits, including higher date rates, lower power consumption, transmission range extension, and more, beyond those of traditional point-to-point communications [4,5].

Various channel coding schemes with DF cooperation were proposed in [6]. Several of these were employed using Turbo codes [7,8] and low-density parity-check (LDPC) codes [9]. Polar codes are new channel codes that can approach the Shannon limit while having low coding complexity [10]. Optimal polar code construction is generally challenging to accomplish. Therefore, many suboptimal polar code construction approaches have been proposed at different levels of computational complexity [11,12,13]. The use of polar codes for cooperative communications has been explored in literature as well; in [14], polar codes were proven to be applicable for degraded wiretap and relay channels, and outperformed competitive counterparts, e.g., optimally designed LDPC codes. In addition, a polar coded-cooperation network (NPCC) with joint successive cancellation (SC) decoding at the destination was proposed in [15]. It was adopted to achieve coded cooperation between two users, and offered network coding to improve the efficiency of the coded cooperation and overall BER performance. However, all of the above schemes were investigated only under a single relay cooperation. In [16], multiple relay selection cooperation was proposed to alleviate multiplex loss through optimal relay selection. The optimization objective was to find the relay with best channel quality for message forwarding. Recently, there had been several relay selection strategies in cooperative communications based on the AF and DF protocols [17,18,19,20]. In [21], a practical code design for a new relay cooperation method called dynamic selective decode-and-forward (D-SDF) was proposed. In this scheme, each relay individually decides the end of the listening phase and the beginning of the cooperation phase based on different source selection rules. In [22], adaptive relay-based SDF cooperation reduced power overhead and achieved optimal outage performance (OP). However, all relays in the decoding set participated in cooperative transmission to obtain optimal interrupt performance, leading to a decline in the spectral efficiency of the entire system. For full collaboration, opportunistic relaying (OR) improved the spectrum efficiency [23]. By selecting an optimal relay to forward messages, the complexity was reduced while diversity gain, which is equivalent to full collaboration, was obtained [24]. In [25,26], the performance of DF-based opportunistic relay selection strategies were studied with cell interference in a multi-cell environment. To solve the error propagation caused by incorrect DF relay decoding, an SDF relay protocol was proposed in [27]; the relay was able to forward source messages that were free of errors. The emergence of the SDF protocol solved the issue of decoding errors in relay nodes while maintaining participation in cooperation [28]. However, all the above schemes assume that the messages are transmitted through relay nodes at any time regardless of the quality of the trunk link, that is, an all-weather relay cooperation scheme. Liu et al. proposed a cooperative relay transmission only when the performance of the trunk link was good [29]. Then, only a link with enough channel gain ratio over the decision threshold was adopted to deliver the messages [30]. However, this may lead to the loss of good diversity in multi-relay cooperation, and can easily cause too much re-transmission in cooperation due to low transmission efficiency and poor energy utility. Finally, factors such as the instantaneous channel state information CSI, OP, signal-to-noise (SNR) boundary, and bit error rate (BER) should be taken into account in optimization of DF protocols.

In the proposed polar coded SDF (PCSDF) scheme, a number of relays are chosen and combined with the optimal CSI compound to optimize the BER performance. According to this conception, efficient polar coded SDF relay selection cooperation is proposed under a cooperative threshold decision derived from the CSI. In addition, the corresponding OP of the proposed relay selection is derived, and the BER with different numbers of relay nodes under the optimal threshold is analyzed. In summary, our main contributions are briefly summed up as follows:

- **Polar codes with specific rates are designed for fading channels.** A new construction approach is proposed using polar codes in fading channels. The information bit and frozen indices are reselected based on the channel states. In addition, an optimally designed SNR is used in code design with fixed data rate. Thus, coding gain is obtained and the complexity is reduced by using the improved polar codes.
- **Optimal selection is obtained for multi-relay cooperation.** The scheme provides a candidate relay set to choose the optimally selected relays and sets a cooperative threshold at the destination to decide whether the relay takes part in cooperative communications. Several relays with good channel gain ratios are chosen and combined in order to exceed the decision threshold for joint cooperative reception. In addition, the OP for different numbers of relays are analyzed theoretically using the proposed PCSDF scheme for more accurate analytical expression.
- **The influence of distance between the source and the optimal relay is numerically analyzed for better performance.** The distance between the source and the optimal relay is a crucial factor in determining overall performance. Through numerical analyses, the relationship of the distance between the source and optimal relay is obtained. The link status associated with the proposed relay position obviously affects the cooperative schemes, which can be adopted in practice.

The remainder of this paper is organized as follows. In Section 2, a multi-relay cooperation system is introduced. Then, a polar encoding construction based on Rayleigh fading channels and PCSDF cooperation is presented in Section 3. In this section, an optimal relay selection is proposed to improve the BER and OP performance. Subsequently, analytical expressions of the optimal SNR design in polar code construction and the OP of the PCSDF scheme are derived in Section 4. In Section 5, simulation results and numerical analyses are presented to verify the good BER and OP performance of our proposed approach, and the resulting complexity is analyzed concisely. Finally, Section 6 concludes the paper.

## 2. System Model of the SDF Cooperation

**Figure 1:**
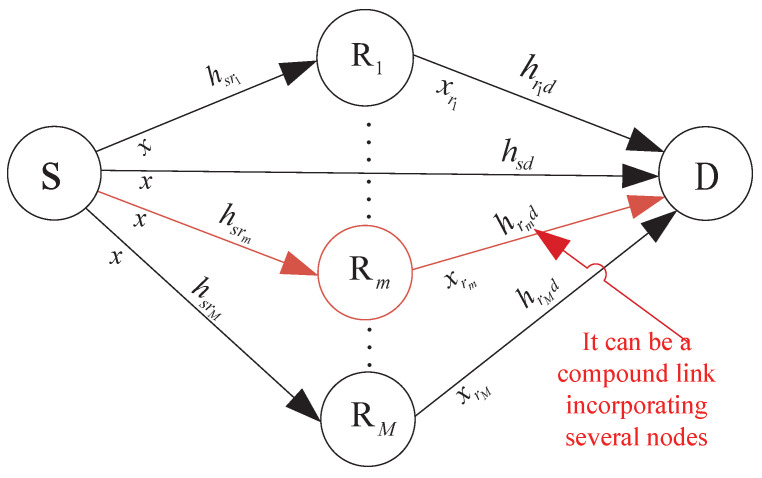
A typical wireless multi-relay system.

A typical wireless multi-relay system at a flat Rayleigh fading channel is shown in Figure 1. It consists of a source node S, several relay nodes R${}_{i}$ (i=1,2,…,N), and a destination node D. The ideal CSI is obtained through channel training. For independent links, e.g., links S-D, S-R${}_{i}$, and R${}_{i}$-D, their channel gains are $|h_{sd}{|}^{2}$, $|h_{sr_{i}}{|}^{2}$, and $|h_{r_{i}d}{|}^{2}$, respectively. The noise is additive white Gaussian noise (AWGN) with zero mean and variance $N_{0}$.

In an SDF cooperative system of poor direct S-D channel quality, the whole transmission is usually divided into two phases, i.e., Phase I and Phase II. In Phase I, the source S broadcasts messages to all relays {R${}_{i}$} and the destination D; in Phase II, either the source node or the selected relay nodes send the messages to destination node, mainly depending on the status of relay channels. Thus, the messages received at the *i*-th relay and the destination are separately expressed as

(1) $$y_{sr_{i}}=\sqrt{P_{s}}h_{sr_{i}}x_{s}+n_{sr_{i}},$$

(2) $$y_{sd}=\sqrt{P_{s}}h_{sd}x_{s}+n_{sd},$$

where $y_{sr_{i}}$ and $y_{sd}$ are the received signals at the *i*-th relay and the destination, respectively; $x_{s}$ and $P_{s}$ are the transmitted signals and the power at source node in Phase I, respectively; $h_{sr_{i}}$ and $h_{sd}$ are the channel fading coefficients of link S-R${}_{i}$ and link S-D, respectively; and $n_{sr_{i}}$ and $n_{sd}$ are the noise of links S-R${}_{i}$ and S-D, respectively.

In Phase II, if the received messages are decoded correctly at the *i*-th relay R${}_{i}$, the decoded signals are re-encoded and re-transmitted to the destination D. Then, the signals received at the destination are expressed as

(3) $$y_{r_{i}d}=\sqrt{P_{r_{i}}}h_{r_{i}d}x_{r_{i}}+n_{r_{i}d},$$

where $x_{r_{i}}$ and $P_{r_{i}}$ are the transmitted signals and power at the *i*-th relay and $n_{r_{i}d}$ and $h_{r_{i}d}$ are the noises and channel coefficients of link R${}_{i}$-D, respectively. Finally, using the maximum ratio combining (MRC) [29] at the destination D, the combined messages are represented as

(4) $$y=ay_{\mathrm{sd}}+\sum_{i\in relay\;set}b_{i}y_{r_{i}d},$$

where *a* and $b_{i}$ are combining coefficients, denoted as $a=\sqrt{P_{s}}h_{sd}^{*}/N_{0}$ and $b_{i}=\sqrt{P_{i}}h_{r_{i}d}^{*}/N_{0}$, respectively. Here, the superscript “*” denotes the conjugate operation.

## 3. Polar Coded SDF Cooperation

### 3.1. Polar Encoding and Decoding

A polar code is specified completely by a parameter pair (N,K,f), where *N* is the code length, *K* is the number of information bits per code word, and *f* is a set of N−K integer indices, which are called frozen bit locations, from 1,2,…,N. Let *W* be a binary discrete memoryless channel (DMC) with an input alphabet x=0,1, output alphabet *y*, and transition probability W(y|x). The channel mutual information with equiprobable inputs, or symmetric capacity, is then defined by

(5) $$I\left(W\right)=\sum_{y\in Y}\sum_{x\in X}\frac{1}{2}W\left(y|x\right)\log\frac{W(y|x)}{\frac{1}{2}W\left(y|0\right)+\frac{1}{2}W\left(y|1\right)},$$

with Barth’s parameters:

(6) $$Z\left(W\right)=\sum_{y\in Y}\sqrt{W(y|0)W(y|1)}.$$

With a code block of length *N*, the channel input and output sequences are denoted by $x_{1}^{N}$ and $y_{1}^{N}$, respectively, with the corresponding vector channel $W^{N}\left(y_{1}^{N}|x_{1}^{N}\right)$.

Then, the procedure of polar coding is summarized as follows.

- **Channel polarization**

Considering the matrix $G_{2}=\left[\begin{array}{cc} 1 & 0\\ 1 & 1 \end{array}\right]$, let $G_{N}=G_{2}^{⊗n}$ be the *n*-th Kronecker power of $G_{2}$, where $n=log_{2}N$. Input bits are denoted by $u_{1}^{N}\in {\left\{0,1\right\}}^{N}$. The vector channel is $W^{N}\left(y_{1}^{N}|u_{1}^{N}\right)=W^{N}\left(y_{1}^{N}|u_{1}^{N}G_{N}\right)$. From $W^{N}\left(y_{1}^{N}|u_{1}^{N}\right)$, a successive cancellation (SC) decoder implicitly defines the bit channel (with i∈[N]) as follows:

(7) $$W_{N}^{\left(i\right)}\left(y_{1}^{N},u_{1}^{i-1}|u_{i}\right)=\sum_{u_{i+1}^{N}}W_{N}\left(y_{1}^{N}|u_{1}^{N}\right).$$

The channel polarization theorem [10] states that $I(W_{N}^{\left(i\right)})$ converges to either “0” or “1” as *N* tends to infinity and the fraction of the noiseless channel tends to $I(W_{N}^{\left(i\right)})$. Then, polar codes with rate R=K/N are constructed by selecting the N−K indexes with the smallest $I(W_{N}^{\left(i\right)})$ or the largest $Z(W_{N}^{\left(i\right)})$ for i∈[N]. These are called the frozen set, denoted as *f*, and the corresponding input bits are set to zero. The complementary unfrozen set of *K* indexes, e.g., $f^{c}$, correspond to information bits.

- **Polar encoding**

**Figure 2:**
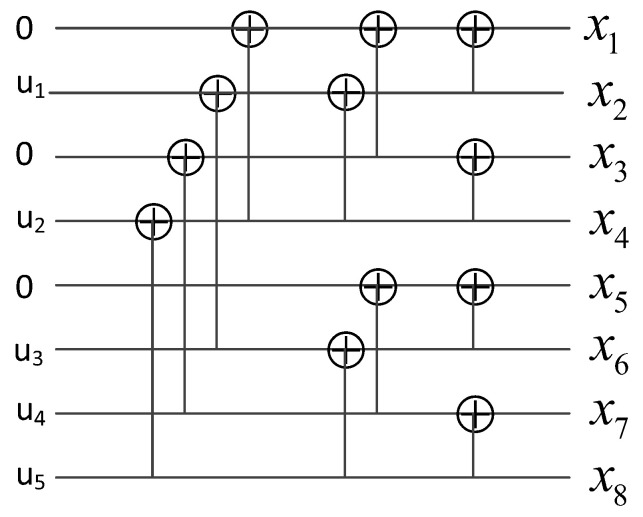
Typical polar encoding in Equation (7) with the systematic parameters (N,K,f)=(8,5,{1,3,5}).

For a polar code (N,K,f), a code word is generated as follows:

(8) $$x_{1}^{N}=u_{f^{c}}G_{N}\left(f^{c}\right)⊕u_{f}G_{N}\left(f\right),$$

where $u_{f^{c}}$ is a *K*-length information bit vector, $u_{f}$ is the frozen information of zero, and $G_{N}$ is the generator matrix of the polar code. An efficient implementation with typical computational complexity O(NlogN) as encoded by (7) is illustrated in Figure 2.

- **Polar successive cancellation (SC) decoding**

In SC decoding, the information bits are estimated as

(9) $${\hat{u}}_{i}=\arg\max_{u_{i}\in \left\{0,1\right\}}W_{N}^{\left(i\right)}\left(y_{1}^{N},u_{1}^{i-1}|u_{i}\right),i\in f^{c}.$$

SC decoding follows essentially the same diagram as in Figure 2 using a decoding operation that resembles one iteration of the existing belief propagation decoding. With a code length *N*, the complexity of the SC decoding is O(NlogN) [3].

- **Construction of polar codes in fading channels**

The polarization phenomenon appears in arbitrary discrete memoryless channels as well [31]. The transmission in a general fading channel is expressed as

(10) y=h·x+n,

where *h* is the channel coefficient of a fading channel, and fits a normal distribution N(0,1). Here, *n* is the AWGN with zero mean and variance $\sigma^{2}$. Due to limitations of the channel estimation, there is a limit of the channel values below which the estimation is unreliable; this is represented as a variable α. For a given value of α, a percentage is calculated as

(11) p=Pr{|h|≤α},

where N×p unreliable observations are performed at the receiver. These observations in return cause poor BER performance. In this case, either a higher SNR or a lower data rate should be applied in order to maintain the desired BER performance. From [32], the relationship between the block error rate $P_{e}$ with data rate *R* and the capacity of the underlying channel I(W) are represented as

(12) $$P_{e}=2^{-2^{\frac{n}{2}+\sqrt{n}Q^{-1}\left(\frac{R}{I(W)}\right)+o\left(\sqrt{n}\right)}},$$

where $Q^{-1}\left(x\right)$ is the reverse function of Q(x), which is represented as $Q\left(x\right)=\frac{1}{\sqrt{2\pi }}\int_{x}^{\infty }e^{-\frac{t^{2}}{2}}dt$. Then, the channel capacity I(W)=C is derived as

(13) $$C=\underset{\left|h\right|}{E}\left\{C(|h\right|\sqrt{\gamma })\},$$

where γ is the SNR and $C(|h|\sqrt{\gamma })$ is defined as

(14) $$\begin{array}{c} C(|h|\sqrt{\gamma })=\int_{-\infty }^{+\infty }\frac{1}{\sqrt{2\pi }}e^{\frac{-(y-|h|\sqrt{\gamma }{)}^{2}}{2}}\log_{2}\frac{2}{1+e^{-2y|h|\sqrt{\gamma }}}dy\\ \;\;\;\;\;\;\;\;\;\;\;\;\;\;\;\;\;+\int_{-\infty }^{+\infty }\frac{1}{\sqrt{2\pi }}e^{\frac{-(y+|h|\sqrt{\gamma }{)}^{2}}{2}}\log_{2}\frac{2}{1+e^{2y|h|\sqrt{\gamma }}}dy \end{array}.$$

The variable $P_{e}$ is concave in terms of the data rate *R* with a fixed I(W). With a data rate, increasing SNR does not yield a significant BER performance improvement at high SNR regions. Instead, a proper method is used to decrease the data rate *R*. By this method, a new polar coding scheme is accomplished to improve BER performance in fading channels. To obtain both good and poor channel indices in polar code construction, a certain amount of channels must be detected.

Finally, a detailed polar coding scheme for fading channels is concluded as follows.

(i) Input a channel fading vector $h=\{h_{1},h_{2},\ldots ,h_{N}\}$, the limit of the channel value α, data rate *R*, code length *N*, *M* and $Q_{s}$, where M=N×p is selected as a tradeoff between BER performance and decreased code rate. Here, $Q_{s}$ is a channel index matrix chosen according to the quality of the bit channels and *M*.
(ii) Count the number of bit channels where $h_{i}$ is less than α, denoted as $N_{rm}$. If $N_{rm}$ is larger than *M*, the actual number of unreliable channel is represented as $M_{i}=M$. Otherwise, $M_{i}=N_{rm}$.
(iii) The number of new frozen bits is expressed as $M_{i}\cdot c$, where *c* is the calculated channel capacity according to the operation SNR = γ and the capacity $C=\underset{\left|h\right|}{E}\left\{C(|h\right|\sqrt{\gamma })\}$ in (13).
(iv) The indices of information bits are $Q_{s}\left(1,\left(K-M_{i}\times c\right)\right)$ and those of frozen bits are $Q_{s}\left[\left(K-M_{i}\times c+1\right),N\right]$. Both of them are ranked in the natural sequence.

### 3.2. Proposed PCSDF Cooperation

In original SDF cooperation, OP performance is limited by the channel quality of the S-R${}_{i}$ link. Whether the relay nodes can decode the source information correctly or not has a crucial impact on the system performance. To reduce error propagation and increase BER performance, an improved PCSDF relay cooperation is proposed in this section. After the relay receives messages from the source in Phase I, the procedure of decoding and forwarding begins. To ensure that the messages are correctly decoded, the channel capacity of link S-R${}_{i}$ is required to be no less than the information transmission rate *V*. Therefore, the set of candidate relay nodes is expressed as

(15) $$\Omega_{k}=\left\{R_{i}:C_{sr_{i}}\geq V\right\},$$

where $C_{sr_{i}}$ is the channel capacity of link S-R${}_{i}$. If the candidate relay set $\Omega_{k}$ is empty, an attempt at direct transmission through link S-D is performed. In this case, the channel capacity of the transmission system is expressed as

(16) $$C_{\mathrm{DRT}}=\frac{1}{2}\log_{2}\left(1+2\gamma_{sd}\right),$$

where $C_{\mathrm{DRT}}$ is the channel capacity of direct link S-D, and $\gamma_{sd}=\frac{P_{s}{\left|h_{sd}\right|}^{2}}{N_{0}}$ is the instantaneous SNR when the destination node D receives the signals from the source node S directly. Furthermore, a relationship $E_{b}/N_{0}=\gamma_{sd}\cdot \log_{2}\left(MI\right)$, exists, where MI is the modulation index, e.g., 2 for binary phase shift keying (BPSK), 4 for quadrature phase shift keying (QPSK), etc. In another situation, if the candidate relay set $\Omega_{k}$ is not empty, a relay node with different instantaneous channel quality from all relay nodes is selected as the *i*-th candidate relay node R${}_{c,i}$ in set $\Omega_{k}$. The SNR of the received signals from the relay node R${}_{c,i}$ is expressed as

(17) $$\underset{\begin{array}{ccc} Subject & to & \end{array}R_{c,i}\in \Omega_{k}}{\gamma_{r_{i}d}=\frac{P_{r_{i}d}{\left|h\right|}^{2}}{N_{0}}},$$

where $\gamma_{r_{i}d}$ is the SNR when destination node D receives signals from the relay node R${}_{i}$, and mainly depends on the transmission power of the relay node and the channel fading coefficient of link $R{}_{i}$-D. When the transmission power of the source node S and each relay node R${}_{i}$ is equal, $R{}_{c,i}$ is only related to the channel fading coefficient $h_{r_{i}d}$.

After the relay node is successful, the ratio of the cooperative capability of R${}_{c,i}$, i.e., $\beta_{r_{c,i}}={\left|h_{r_{c,i}d}\right|}^{2}$ with instantaneous channel gain of link S-D, i.e., $\beta_{sd}={\left|h_{sd}\right|}^{2}$ is obtained at the destination. With an efficient information rate, or code rate τ, used in the DF cooperation, the OP $P_{DF}$ can be expressed as

(18) $$\begin{array}{c} P_{DF}\left(\gamma_{sr_{i}},\tau \right)=P\left(I_{DF}<\tau \right)\\ =P\left[{\left|h_{sr}\right|}^{2}<\left(2^{2\tau }-1\right)/\gamma_{sr_{i}}\right]+P\left[{\left|h_{sr}\right|}^{2}\geq \left(2^{2r}-1\right)/\gamma_{sr_{i}}\right]\\ =P[{\left|h_{sd}\right|}^{2}+{\left|h_{r_{i}d}\right|}^{2}<\left(2^{2\tau }-1\right)/\gamma_{sr_{i}}]\\ ∼\left(2^{2\tau }-1\right)/\left(\sigma_{sr_{i}}^{2}\cdot \gamma_{sr_{i}}\right) \end{array},$$

where $I_{DF}$ is the channel capacity of the DF cooperation system and is represented as $I_{DF}=\min_{i}\left\{I_{sr},I_{sr_{i}d}\right\}$. Thus the cooperation threshold θ is in proportion to the reverse of the SNR $\gamma_{sr_{i}}$; it can be derived from (18) and expressed as $\theta =\left(2^{2\tau }-1\right)/\gamma_{sr_{i}}$.

Subsequently, the ratio is compared with the cooperation threshold θ. If $\beta_{r_{c,i}}/\beta_{sd}>\theta$, the quality of the link including the selected relay is optimal. Thus, $R{}_{c,i}$ can be used to forward messages. The received signals in the destination D are then expressed as

(19) $$y_{r_{c,i}d}=h_{r_{c,i}d}\sqrt{P_{r_{c,i}}}x_{r_{c,i}}+n_{rd_{c,i}}.$$

If $\beta_{r_{opt}}/\beta_{sd}\leq \theta$, the channel quality of the chosen link R${}_{opt}$-D is rather poor. Thus, the relay $R_{opt}$ is frozen and the source S transmits messages to the destination D. The received signals are then expressed as

(20) $$y_{sd}^{\left(2\right)}=h_{sd}^{\left(2\right)}\sqrt{P_{s}}x_{s}+n_{sd}^{\left(2\right)}.$$

Finally, the received signals from all links are combined together using the maximum ratio combination (MRC) method [29] at the destination. The combination of all messages is expressed as

(21) $$y=\left\{\begin{array}{c} a_{1}y_{sd}^{\left(1\right)}+a_{2}y_{rd_{opt}},\;\;\;\;\;\beta_{r_{opt}}/\beta_{sd}>\theta \\ a_{1}y_{sd}^{\left(1\right)}+a_{2}y_{sd}^{\left(2\right)},\;\;\;\;\;\beta_{r_{opt}}/\beta_{sd}\leq \theta \end{array}\right.,$$

where $a_{1}$ and $a_{2}$ are the combining coefficient and $y_{sd}^{\left(1\right)}$ equals $y_{sd}$ in (2). The instantaneous SNR at the destination is expressed as

(22) $$\gamma =\left\{\begin{array}{c} \gamma_{sd}^{\left(1\right)}+\gamma_{{}_{rd_{opt}}},\;\;\;\;\;\;\;\;\;\;\;\;\;\beta_{r_{opt}}/\beta_{sd}>\theta \\ \gamma_{sd}^{\left(1\right)}+\gamma_{sd}^{\left(2\right)},\;\;\;\;\;\;\;\;\;\beta_{r_{opt}}/\beta_{sd}\leq \theta \;\; \end{array}\right.,$$

where the instantaneous SNR in Phase I with link S-D is $\gamma_{sd}^{\left(2\right)}$, that in Phase II is $\gamma_{sd}^{\left(2\right)}$, and $\gamma_{rd_{opt}}$ is the instantaneous SNR of link R${}_{opt}$-D. Then, the maximum transmission rate of the entire system using this scheme is expressed as

(23) $$C_{\mathrm{PCSDF}}=\left\{\begin{array}{c} \frac{1}{2}\log_{2}\left(1+\gamma_{sd}^{\left(1\right)}+\gamma_{rd_{opt}}\right),\;\;\;\;\;\beta_{r_{opt}}/\beta_{sd}>\theta \\ \;\frac{1}{2}\log_{2}\left(1+\gamma_{sd}^{\left(2\right)}+\gamma_{sd}^{\left(1\right)}\right),\;\;\;\;\;\beta_{r_{opt}}/\beta_{sd}\leq \theta \end{array}\right..$$

In the above scheme with only one relay link with a sufficient channel gain ratio (i.e., the corresponding instantaneous SNR) is adopted to deliver the messages. Thus, the diversity cannot be utilized, and there is a higher chance of retransmission. An improved version of the above scheme can be realized by combining several messages from several merged R${}_{i}$-D links together to generate equivalent and strengthened hybrid signals with instantaneous SNR larger than the cooperation threshold. This can be easily implemented using the maximum ratio combination (MRC) technique, where their SNR can accumulate linearly. In addition, for the purposes of lower computational complexity, a maximum of five path signals can be chosen and merged together for possible signal strengthening to exceed the cooperation threshold. Using this method, the signal diversity is fully adopted with a significant reduction in retransmission and better transmission efficiency.

**Figure 3:**
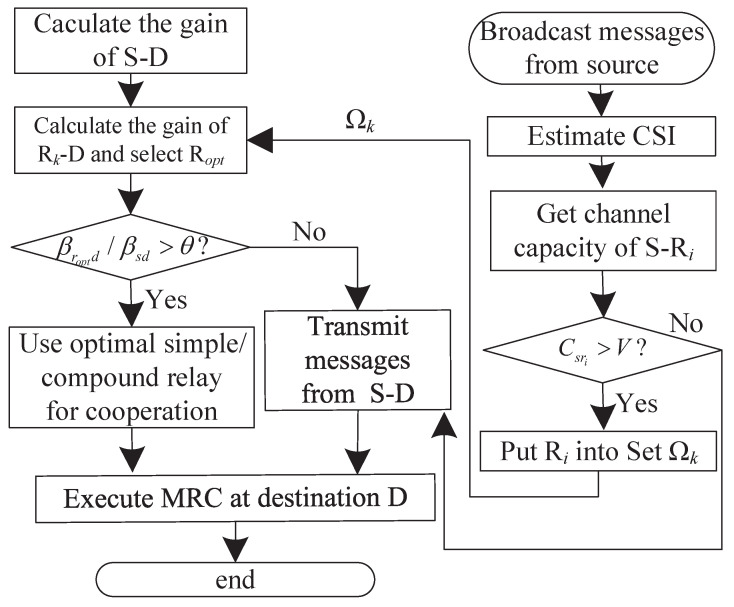
Flow chart of the proposed SDF cooperation scheme.

In summary, the main procedures of the proposed PCSDF cooperation approach are shown in Figure 3 and Table 1.

**Figure 4:**
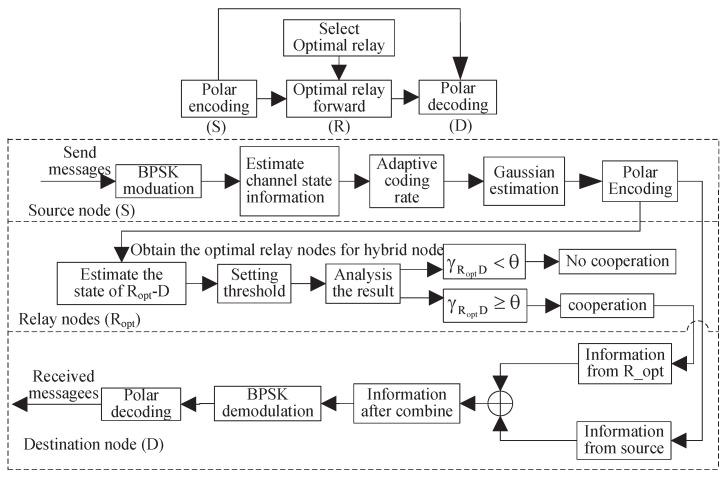
Block diagram of the PCSDF cooperation scheme.

Based on the above description, the block diagram of the whole PCSDF cooperation scheme can be designed, as shown in Figure 4.

## 4. Theoretical Analysis of the Proposed PCSDF Cooperation Scheme

### 4.1. Analysis of Optimal Design-SNR in Proposed Scheme

For a system with a design-SNR γ and data rate *R* in AWGN channels, the polar code can be constructed by the design-SNR [31]. The construction changes with different design-SNR. The optimal design-SNR can produce good BER performance for a fixed data rate *R* at a range of SNRs [11].

For a fixed data rate *R*, the required SNR γ to fit this data rate *R* can be obtained as

(24) $$\gamma =C^{-1}\left(R\right),$$

where $C^{-1}\left(x\right)$ is the inverse function of C(x) in (13). The required SNR for a data rate *R* is denoted as $\gamma_{R}$. For a fixed data rate *R*, $\gamma_{R}$ is the optimal design-SNR for the construction of the polar codes.

### 4.2. Analyses of Outage Probability in Proposed Scheme

Outage probability (OP) is defined as the probability of transmission failure, which is one of the most crucial measures to evaluate the entire wireless communications setup. A transmission interruption occurs when the link capacity cannot attain the required user rate. Therefore, for the PCSDF scheme, an interruption occurs in the three situations described below.

(i) The outage probability $P_{1}$ that refers to that the source S re-transmits messages to the destination D when $\Omega_{k}=\emptyset$.
(ii) The outage probability $P_{2}$ relates to the failure of the optimal relay when $\Omega_{k}\neq \emptyset$ and $\beta_{r_{opt}}/\beta_{sd}\leq \theta$.
(iii) The outage probability $P_{3}$ of the optimal relay is used to forward messages when $\Omega_{k}\neq \emptyset$ and $\beta_{r_{opt}}/\beta_{sd}>\theta$.

Thus, the OP of the entire system is expressed as

(25) $$P=P_{1}+P_{2}+P_{3}.$$

Subsequently, the above three situations can be analyzed as follows.

Situation I. When only link S-D is used to transmit messages, an interruption occurs until the channel capacity $C_{sd}$ is less than the message transmission rate *V*; the probability of $\Omega_{k}=\emptyset$ is calculated as

(26) $$P\left(\Omega_{k}=\emptyset \right)=\prod_{i=1}^{N}\mathrm{Pr}(C_{sr_{i}}<V).$$

Thus, the OP can be expressed as

(27) $$\begin{array}{c} P_{1}=P\left(\Omega_{k}=\emptyset \right)P\left(C_{sd}<V\right)\\ \;\;\;\;=\prod_{i=1}^{N}\mathrm{Pr}\left(C_{sr_{i}}<V\right)\mathrm{Pr}\left(C_{sd}<V\right)\\ \;\;\;\;=(1-\exp(-\frac{2^{V}-1}{\Gamma_{sr}}{))}^{N}\left(1-\exp\left(-\frac{2^{V}-1}{\Gamma_{sd}}\right)\right) \end{array},$$

where $\Gamma_{sr}$ and $\Gamma_{sd}$ are the average SNR of links S-R and S-D, respectively.

Situation II. When *K* relays are selected to be the candidate relays from all *M* relays, the probability of $\Omega_{k}\neq \emptyset$ is expressed as

(28) $$\begin{array}{c} \mathrm{Pr}\left(\Omega_{k}\neq \emptyset \right)=C_{M}^{K}\prod_{i\in \Omega_{k}}\mathrm{Pr}(C_{sr_{i}}\geq V)\prod_{i\notin \Omega_{k}}\mathrm{Pr}(C_{sr_{i}}<V)\\ \;\;\;\;\;\;\;\;\;\;\;\;\;\;\;\;\;=C_{M}^{K}\exp{\left(-\frac{2^{V}-1}{\Gamma_{sr}}\right)}^{K}{\left(1-\exp\left(-\frac{2^{V}-1}{\Gamma_{sr}}\right)\right)}^{M-K} \end{array},$$

where $C_{M}^{K}=\frac{M!}{K!(M-K)!}$. Then, the probability of $\beta_{r_{opt}}/\beta_{sd}\leq \theta$ can be computed as

(29) $$\begin{array}{c} \mathrm{Pr}(\beta_{r_{opt}}/\beta_{sd}\leq \theta )=\mathrm{Pr}(P_{r}|h_{rd_{opt}}{|}^{2}/N_{0}\leq \theta P_{s}{\left|h_{sd}\right|}^{2}/N_{0})\\ \;\;\;\;\;\;\;\;\;\;\;\;\;\;\;\;\;\;\;\;\;\;\;\;=\mathrm{Pr}(\gamma_{rd_{opt}}\leq \theta \gamma_{sd})\\ \;\;\;\;\;\;\;\;\;\;\;\;\;\;\;\;\;\;\;\;\;\;\;\;=\prod_{i=1}^{k}\mathrm{Pr}(\gamma_{r_{i}d}<\theta \gamma_{sd})\\ \;\;\;\;\;\;\;\;\;\;\;\;\;\;\;\;\;\;\;\;\;\;\;\;=(1-\exp(-\frac{\theta \gamma_{sd}}{\Gamma_{rd_{opt}}}{))}^{k} \end{array}.$$

With an actual information transmission rate *V*, $C_{\mathrm{PCSDF}}$ is less than *V* when the optimal relay is out of work. This can be written as $\frac{1}{2}\times \log_{2}\left(1+\gamma_{sd(1)}+\gamma_{sd(2)}\right)<V$. Thus, the OP $P_{2}$ is expressed as

(30) $$\begin{array}{c} P_{2}=\mathrm{Pr}\left(\Omega_{k}\neq \emptyset \cap \beta_{r_{opt}}/\beta_{sd}\leq \theta \right)\\ \;\;\;\;\;\;\;\times \mathrm{Pr}(\gamma_{sd(1)}+\gamma_{sd(2)}<2^{2V}-1)\\ \;\;\;\;=C_{M}^{K}\exp{\left(-\frac{2^{V}-1}{\Gamma_{sr}}\right)}^{K}{\left(1-\exp\left(-\frac{2^{V}-1}{\Gamma_{sr}}\right)\right)}^{M-K}\\ \;\;\;\;\;\;\times (1-\exp(-\frac{\theta \gamma_{sd}}{\Gamma_{rd_{opt}}}{))}^{k}\left(1-\exp\left(-\frac{2^{2V}-1}{\gamma_{sd(1)}+\gamma_{sd(2)}}\right)\right) \end{array}.$$

Situation III. When the optimal relay is selected to take part in cooperation, the channel capacity of the system can be rewritten as $C_{\mathrm{PCSDF}}=\frac{1}{2}\times \log_{2}\left(1+\gamma_{sd(1)}+\gamma_{rd_{opt}}\right)$. Similarly, an interruption occurs when $C_{\mathrm{PCSDF}}$ is less than information transmission rate *V*, i.e., $C_{\mathrm{PCSDF}}<V$. This can be rewritten as

(31) $$\gamma_{sd(1)}+\gamma_{rd_{opt}}<2^{2V}-1.$$

Thus, the OP $P_{3}$ can be calculated as

(32) $$\begin{array}{c} P_{3}=\mathrm{Pr}\left(\Omega_{k}\neq \emptyset \right)\mathrm{Pr}\left(\beta_{r_{opt}}/\beta_{sd}>\theta \right)\\ \;\;\;\;\;\;\;\times \mathrm{Pr}(\gamma_{sd(1)}+\gamma_{rd_{opt}}<2^{2V}-1)\\ \;\;\;\;=C_{M}^{K}\exp{\left(-\frac{2^{V}-1}{\Gamma_{sr}}\right)}^{K}{\left(1-\exp\left(-\frac{2^{V}-1}{\Gamma_{sr}}\right)\right)}^{M-K}\\ \;\;\;\;\;\;\;\times \left(1-(1-\exp(-\frac{\theta \gamma_{sd}}{\Gamma_{rd_{opt}}}{))}^{K}\right)\left(1-\exp\left(-\frac{2^{2V}-1-\gamma_{rd_{opt}}}{\gamma_{sd(1)}}\right)\right) \end{array}.$$

According to the above analysis, the interrupt probability of the proposed PCSDF scheme is proportional to the cooperation threshold θ when the information transmission rate *V* and all channel conditions are known. As θ increases, the number of optimal relays participating in cooperative communication decreases, which is accompanied by an increase in interruption probability for the whole system.

## 5. Numerical Simulations and Analysis of Results

### 5.1. BER Performance of Polar-Coded Cooperation Transmission over Fading Channels

**Figure 5:**
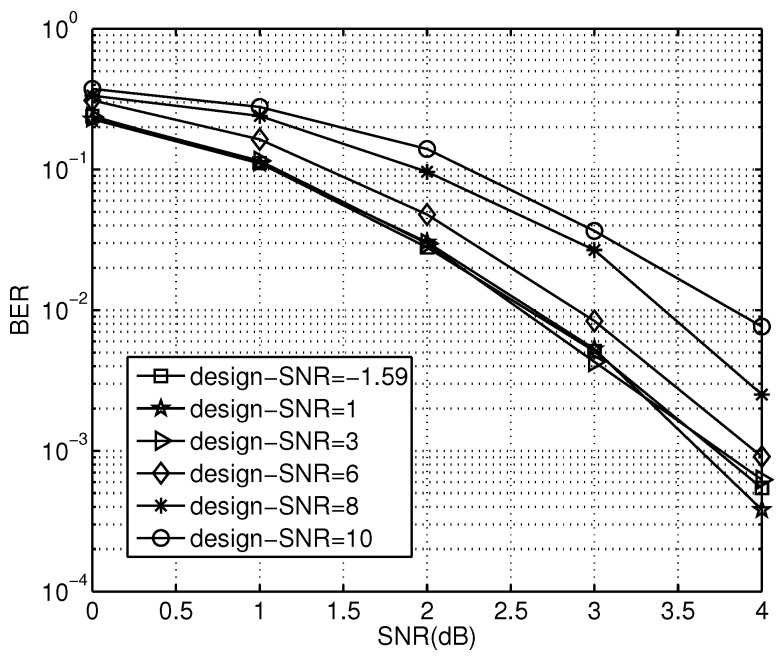
BER performance under different design-SNRs.

To verify the effectiveness of our scheme, polar coded cooperation transmissions with channel fading factor and design-SNR selection are carried out in simulations. The main simulation parameters are set as follows: N=512, R=0.5, α=0.2, and M=32. The BER performance of polar codes using different design-SNR settings are shown in Figure 5. In Figure 5, the BER curves represent the polar codes constructed at different design-SNRs. At high design-SNRs, the BER performance degrades with increasing design-SNR. For instance, there is a performance gain of about 1.3 dB in the scheme from 1 dB to 10 dB design-SNR at BER of $10^{-2}$.

Design-SNR plays an important role in polar codeword construction. The proper design-SNR can improve the code performance significantly. In Figure 5, the code performance decreases with increasing design-SNR. This can be explained as follows. A proper design-SNR is suited to the requirements of polar code construction. Therefore, the channel polarization is improved by the matched design-SNR, and the code performance is optimized.

**Figure 6:**
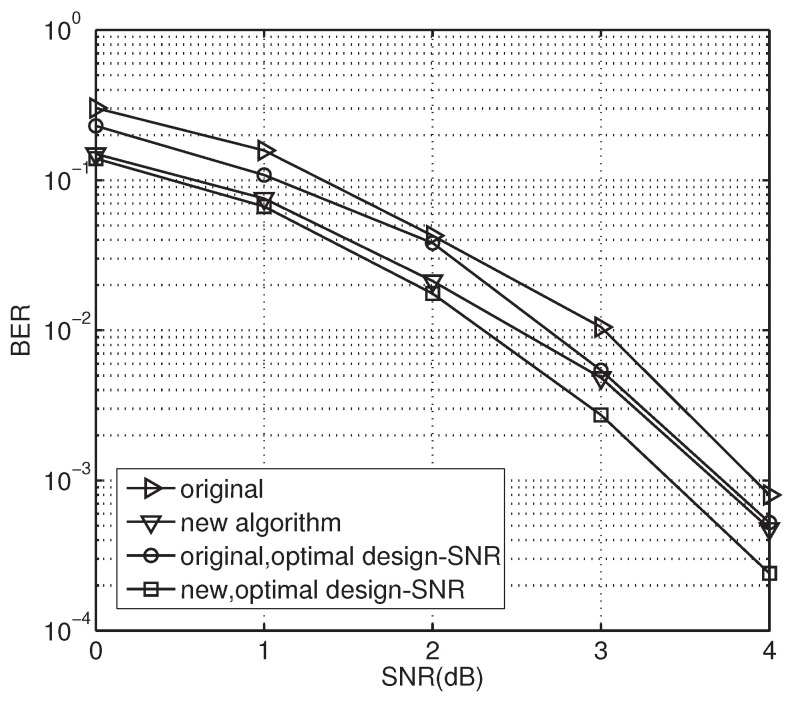
BER performance of the proposed polar codes over fading channels.

The BER performance of polar codes with different constructions is shown in Figure 6, presenting the BER performances of original and new constructions using an optimal design-SNR or not, respectively. As a result, an optimal design-SNR is critical for all construction schemes to ensure that good polar codes are generated. The BER performance is greatly improved by our code construction approach. Even without optimal design-SNR selection, the proposed scheme outperforms the original one by about 0.5 dB at a BER of $10^{-3}$. In addition, it further improves BER performance by about 0.6 dB when using the optimal design-SNR. The performance gain mainly results from the use of the optimal design-SNR in the construction of the polar codes. Then, an optimized design-SNR can be calculated and suited to the requirements of polar code construction for better performance.

### 5.2. Simulations and Analyses of the Proposed PCSDF Cooperation

**Figure 7:**
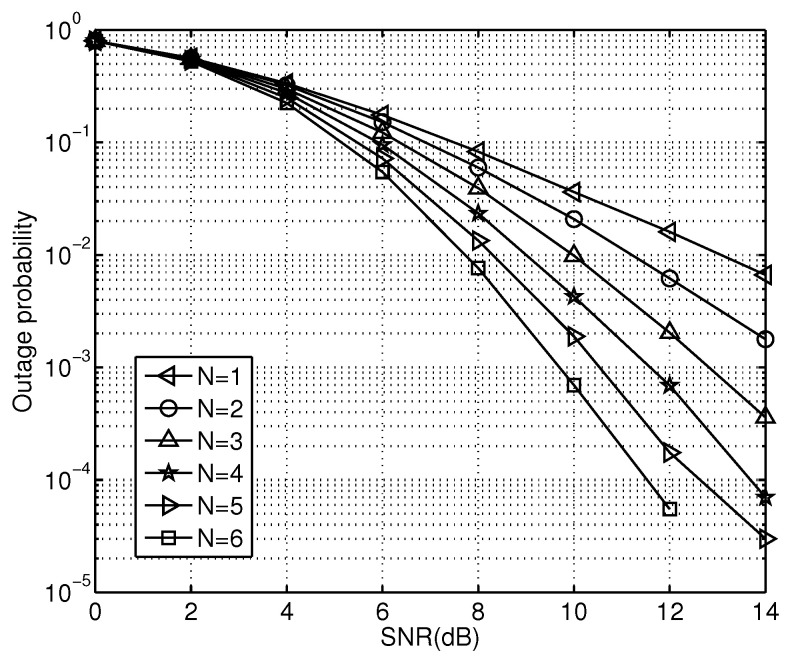
The OP curves of different number relays with a cooperative threshold θ=0.01.

In this section, experiment results are simulated to verify our analysis and evaluate the performance of the proposed PCSDF relay strategy. A multiple-relay cooperative communication model is adopted in simulations, and the parameters are set as follows: the information bit of a code word N=512, and R=0.5. Each channel is an independent Rayleigh channel, and the BPSK modulation is adopted. The transmission power of the source node equals that of each relay node. Figure 7 shows the OP curve of the cooperative system with different relay nodes with a collaboration threshold of 0.01.

In Figure 7, the OP of the proposed PCSDF system decreases as the number of the relays increases. This phenomenon can be explained as follows: as the number of relay nodes increases, the probability of relays correctly decoding the messages increases; thus, the performance of the selected optimal relay from all candidate relays is improved. Obviously, the OP of the proposed scheme decreases as the number of relay nodes increases. On the other hand, the OP decreases as the SNR increases. It can be observed that the SNR of the signals has an effect on the OP of the system. The instantaneous SNR received at the destination node increases as the transmitted SNR increases. Therefore, the OP decreases as the SNR increases.

**Figure 8:**
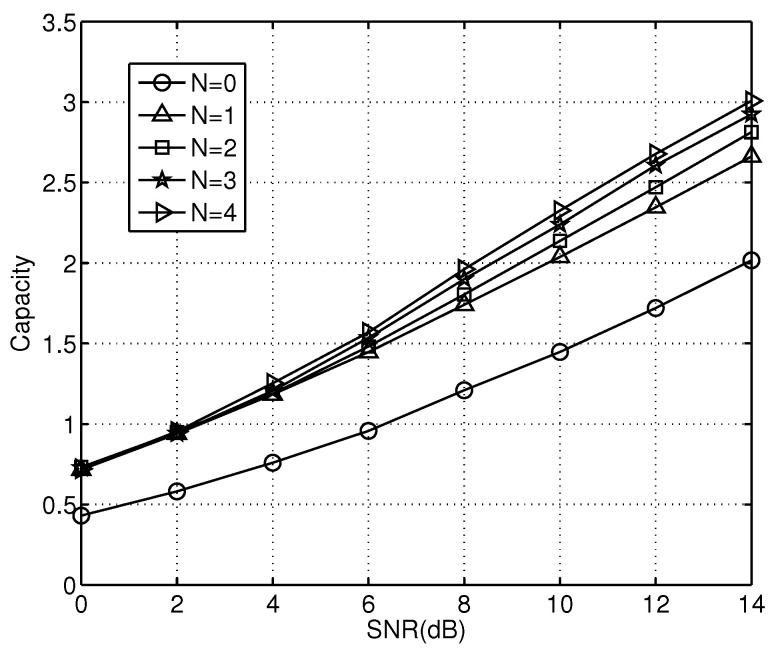
Channel capacity with different numbers of relays, θ=0.01.

The maximum channel capacity of the system is an important indicator for measuring the performance of a system. Equation (22) provides the theoretical channel capacity expression of the proposed cooperation system. The channel capacity curves with no relay nodes and with different numbers of relay nodes are shown in Figure 8.

The results show that the channel capacity of the cooperative relay system is much larger than that of the simple direct transmission system. As the number of relay nodes increases, the channel capacity of the system gradually increases as well. In the case of a channel capacity of two bits/s, the four-relay system has an additional gain of 2 dB compared to the single-relay system. In our simulations with different numbers of relays, the channel capacity of our scheme with relays outperforms that without any relay. In addition, the differences in the proposed scheme with different numbers of relays is trivial. The introduction of relays greatly improves the channel capacity. Therefore, the channel capacity increases as the number of relays increases.

**Figure 9:**
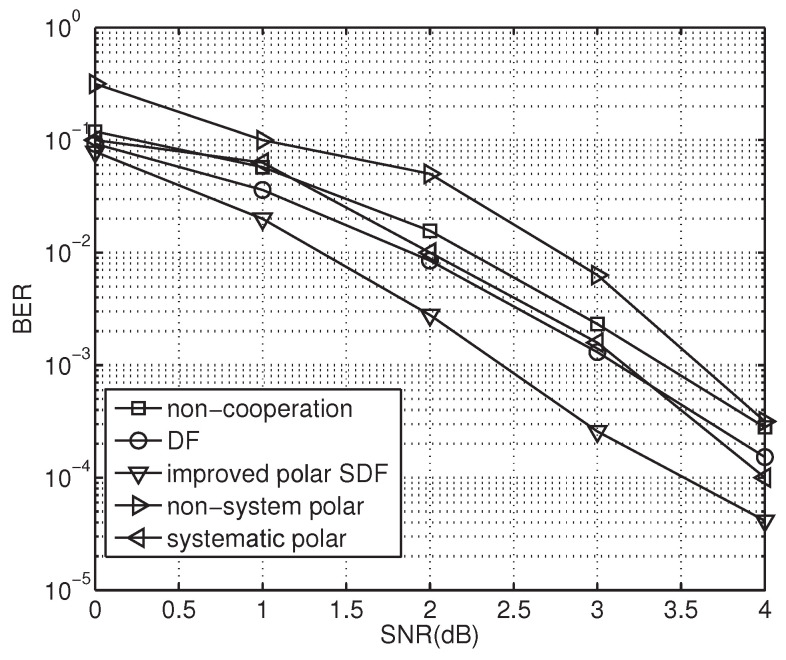
BER performance of different schemes.

Figure 9 shows the BER performance curves for the PCSDF scheme and other relay cooperation schemes. It can be seen from the figure that the performance of the scheme is much better than those of other existing schemes. In the PCSDF scheme, the received messages are forwarded only when the optimal relay channel quality is good. Through this phenomenon, error propagation is reduced and the performance of system is increased. A gain of about 0.9 dB and 0.8 dB is achieved compared with the non-cooperative and standard SDF schemes at a BER of $10^{-3}$, with an approximate performance gain of 1.0 dB and 0.7 dB, respectively, at a BER of $10^{-2}$. This phenomenon can be explained as follows. The proposed scheme uses an efficient channel capacity approaching the polar code to assist the SDF, making for better performance. Moreover, it adopts a compound relay link to exceed the cooperative threshold in order to reduce the chance of re-transmission, achieving better performance and less latency.

**Figure 10:**
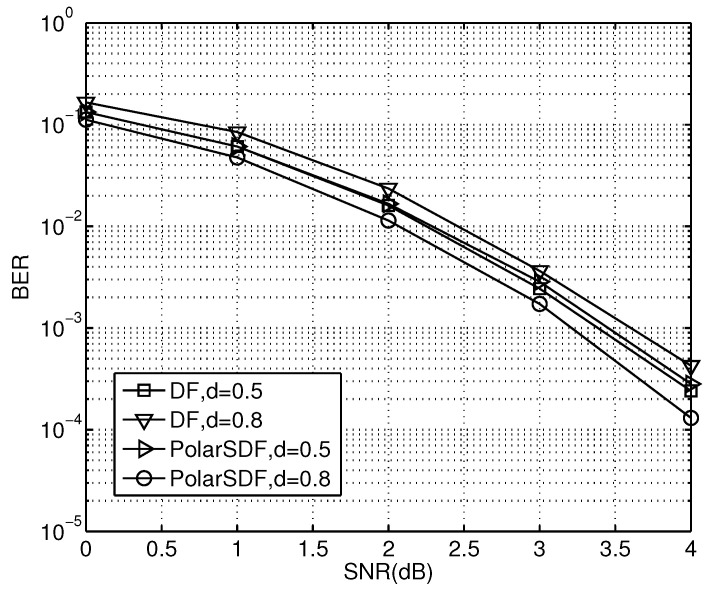
The BER performance of the proposed schemes with different S-R${}_{opt}$ distances.

Furthermore, the distance of link S-R${}_{opt}$ has a deterministic impact on the performance of the relay system. In our simulations, the distance of link S-D is normalized as 1. The distance of link S-R${}_{opt}$ is chosen as a coefficient in proportion to the normalized coefficient of link S-D, and is less than 1. We let the distance of link S-R${}_{opt}$ be *d*, and the distance of link R${}_{opt}$-D be (1−d); then, the BER performance of the PCSDF scheme with different distances *d* is as shown in Figure 10.

The gain increases with the distance *d*. For the PCSDF scheme, there is a gain of about 0.3 dB at a *d* of 0.8, compared to a gain of of 0.5 dB at a BER of $10^{-3}$. The proposed scheme has about 0.5 dB gain compared to the DF one at a BER of $10^{-2}$ and *d* of 0.8. Thus, the BER performance is increased as the distance of link R${}_{opt}$-D decreases. This phenomenon can be explained as follows. This scheme selects the best equivalent relay to forward the decoded messages in the SDF cooperation. With a short distance between the best relay and the destination, the cooperation ability of the former is decreased due to the ease of its absence in cooperation, which is caused by the equalization of the diversity gains and spectrum efficiency. Then, the priority of spectrum efficiency is obtained at the cost of acceptable BER loss. On the contrary, the cooperative participation of the relay is important for large diversity gains and low BER when there is a long distance between the best relay and the destination. In addition, the error propagation, which usually appears in the DF scheme, can be effectively avoided. Only the best relay forwarding procedure can ensure that the received information at the relay nodes is sent to the destination node effectively. Thus, a shorter distance between the best relay and the destination leads to lower error probability or better BER performance. Therefore, in the multi-relay system, a relay located near the destination node can obtain much better performance than other counterparts.

**Figure 11:**
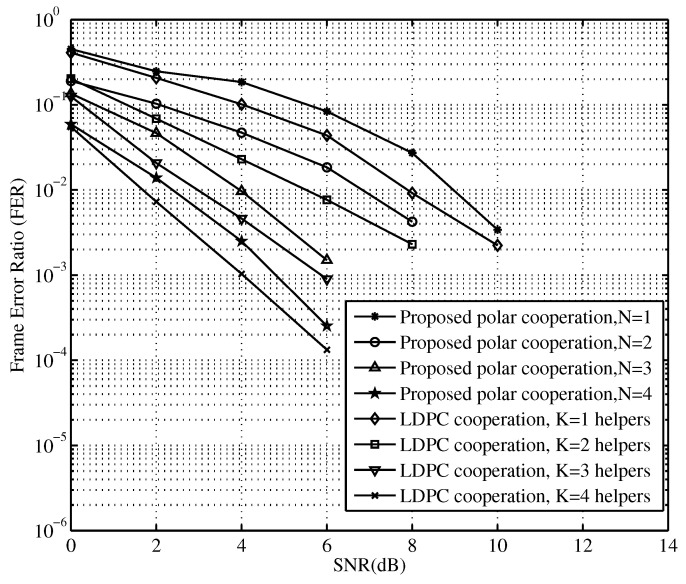
The FER performance of the proposed schemes with different numbers of relays.

To compare the obtained results with recent research, we compare the frame error rate (FER) of our polar coded cooperation scheme with that of the LDPC coded one under different relay numbers. The simulation parameters are configured as follows: code length N=4028, code rate R=0.5, α=0.2, and M=32 for the optimized polar encoding and SC decoding. The FER performances of polar codes using different average SNRs per link are shown in Figure 11. In Figure 11, the FER curves represent the constructed polar codes with cooperation and the counterpart of the LDPC coded cooperation [33]. At high average SNRs per link, the FER performance of our scheme is almost the same as that of the LDPC coded cooperation with the increased SNR. For instance, our scheme has a performance gap of only 0.3–2 dB compared to those of counterparts at SNRs from 0 dB to 12 dB.

In Figure 11, while our scheme exhibits slightly poor performance, it obtains both low complexity and low latency. The performance difference can be explained from two viewpoints, as follows. First, the channel codes adopted in the coded cooperation mean that in the comparison experiments, the LDPC cooperation counterpart uses a rather long code length of 4096 with code rate of 1/2 in cooperation. The constructed of the codewords is improved by using optimized degree profiles for optimal code performance. In addition, for efficient decoding, the belief propagation (BP) algorithm can be adopted for much better performance at the cost of higher complexity and larger latency. In our scheme, however, we use the polar code with simple and non-complex SC decoding at the cost of performance loss. Generally, polar codes are superior in performance to LDPC codes at short code lengths of less than 1000. Thus, they possess much better performance in the case of short codewords. In this experiment, however, due to the moderate code length, LDPC obtains a little better performance at the cost of more complexity and latency. The second aspect is the cooperation strategy used in our scheme. We mainly set a cooperative threshold to accumulate as little relaying as possible in the cooperation in order to guarantee minimum requirements for relay forwarding. This saves transmission energy and is very suitable for use in energy-constrained wireless IoT systems. Fortunately, the performance gap is rather low and tolerant in practice. Although our scheme exhibits somewhat poor performance, the computational complexity is comparably low and the implementation is much easier, making it much more suited for use in hardware resource-limited IoT devices. terminals, etc.

### 5.3. Computational Complexity Analysis of the Proposed PCSDF Cooperation

The computational complexity of the proposed PCSDF cooperation mainly includes two parts, i.e., the encoding and decoding of the polar code and the cooperative signal processing. With a code length *N* and information bit length *M* in a codeword, the complexity of the polar successive cancellation (SC) decoding is provided by O[N·log(M)]. In the cooperative signal processing, with an average of η successful forwarding and 1-η retransmission along with an average of *t* relay participating the compound relay node, there are 4ηt multiplications and 2t additions for each signal sample of successful forwarding, by (19) and (21), respectively. Otherwise, retransmission is performed and an alternative 4ηt multiplications and 2t additions for each signal sample retransmission are determined by (20). The computational complexity of CSI estimation is fixed as $\beta_{r_{opt}}$ and $\beta_{sd}$ in the cooperative transmissions, with one division and one comparison in (21) and (22), respectively. The complexity of the cooperative threshold θ is one exponential calculation, one subtraction, and one division, respectively, according to () and the corresponding explanations. In addition, the threshold is directly calculated in the polar SC decoding after the combination of MRC in the forwarding procedure. The core procedure of multi-path combination to generate a compound link is performed and organized to exceed the threshold using the powerful destination. The destination notifies the proper relay to participate in cooperation for the compound relay with low-cost limited feedback.

## 6. Conclusions

In this paper, an improved PCSDF scheme is proposed with cooperative capability as the decision threshold. Optimal relay selection and OP are suggested and analyzed. The optimization of the cooperative scheme is performed with polar code construction by an optimal design-SNR and a new collaborative construction for more coding gain, which endows it with better BER and OP performance and less computational complexity. In addition, an appropriate cooperative threshold is derived to achieve optimal relay location. When the optimal relay is close to the destination node, it obtains better BER performance in the proposed cooperative scheme. Then, it can be applied in optimal relay position selection for better cooperation. Finally, the channel capacity of the entire cooperative system with different numbers of relays is provided, and a larger information rate can be obtained in the proposed scheme to approach the channel capacity. In summary, the proposed scheme uses the efficient channel capacity approaching the polar codes to assist the multi-relay SDF for better performance. Moreover, it adopts a compound relay link to exceed the cooperative threshold in order to reduce the chance of re-transmission for better performance, lower complexity, and less latency. Therefore, the proposed PCSDF scheme can be effectively adopted in multi-relay cooperative networks in practice for good OP and BER performance.

In addition, there are other possible directions for future improvement of the optimal coded relay selection. First, polar codes with any feasible length, rather than a power of 2, can be constructed with distributed decoding for better application in coded cooperation. Second, DF or SDF cooperation used in this paper; coded cooperation (CC), soft-metric forwarding (SMF), compressed forwarding (CF), and even hybrid combinations of the above schemes can be used in the possible coded cooperation for even better performance. Finally, power allocation can be merged into the cooperation for joint optimization of the transmission resources, which could represent a promising and deep level cross-layer communication optimization in the next generation satellite based 6G IoT systems.

## Figures and Tables

**Table 1:** Procedures of the proposed SDF cooperation scheme.

Procedures of the improved PCSDF cooperation scheme.
Step (1). First, The source node broadcasts messages to the destination and all relays. Then they estimate $h_{sd}$ and $h_{sr_{i}}$, respectively, to obtain the gain of link S-D $\beta_{sd}$ and the capacity of the relay channel $C_{sr_{i}}$.
Step (2). Compare the capacity of the relay channel $C_{sr_{i}}$ with the information transmit rate *V*. if $C_{sr_{i}}\geq V$, the relay $R_{i}$ is selected into the candidate relay set $\Omega_{k}$. Then, goto step (3).
Step (3). If $\Omega_{k}$ is empty, the source transmits messages to the destination. Otherwise, the optimally selected relay $R_{opt}=\underset{R_{i}\in \Omega_{k}}{\arg\max}\left\{\left|h_{r_{i}d}\right|\right\}$ are chosen from the candidate relay set $\Omega_{k}$. Here, $R_{opt}$ can be a simple relay or a compound one composed of several relays. And the latter is jointly combined by several relays for the same channel coefficient with the maximum ratio combination (MRC) criterion. Subsequently, the cooperative capacity of the optimal relay $\beta_{r_{opt}}$ is obtained. Then, goto step (4).
Step (4). Compare the relationship between $\beta_{r_{opt}}$ and $\beta_{sd}$. If $\beta_{r_{opt}}/\beta_{sd}>\theta$, the optimal relay forwards source messages. Otherwise, the optimal relay keeps silent in the whole process.
